# Three-Dimensional (3D) Printing for Left Atrial Appendage Occlusion Device Sizing: A Systematic Review and Comparative Analysis

**DOI:** 10.7759/cureus.82043

**Published:** 2025-04-10

**Authors:** Jasneel S Kahlam, Olga V Savinova, Don D Shamilov, John Tran, Jodie Borgmann, Trinh Tran, Nathan Jean, David F Lo

**Affiliations:** 1 Internal Medicine, Stony Brook Southampton, Southampton, USA; 2 Basic Sciences, New York Institute of Technology, College of Osteopathic Medicine (NYITCOM), Old Westbury, USA; 3 Medicine, American Preventative Screening and Education Association, Stratford, USA

**Keywords:** 3d ct, 3d printing, 3d tee, additive manufacturing, left atrial appendage occlusion, rapid prototyping

## Abstract

In recent years, the clinical application of three-dimensional (3D) printing technology has shown great potential in surgical planning. The left atrial appendage (LAA) is a key site for thrombus formation in nonvalvular atrial fibrillation (NVAF), increasing the risk of stroke. One recent application of this technology is for LAA occlusion, a procedure used to seal the LAA and prevent the formation of systemic thromboembolism associated with NVAF in patients with contraindications for oral anticoagulation therapy.

In this systematic review, the use of 3D printing for device sizing during the planning of LAA occlusion procedures was evaluated through a literature search using the following keywords: ("Left atrial appendage" OR "LAA" or “intra-atrial mass” OR “left atrial mass” ) AND ("3D printing" OR "additive manufacturing" OR "rapid prototyping" OR "computer-aided design" OR "CAD" OR "bioprinting") AND ("occlusion" OR "closure" OR "device sizing" OR "atrial fibrillation").

After data extraction, 16 studies reported using 3D printing, prospectively or retrospectively, to estimate the accuracy of LAA device sizing and/or deployment. These studies demonstrated that 3D-printed models can improve anatomical measurements and allow for improved device sizing and implantation compared to standard-of-care imaging-assisted procedural planning.

Of the articles included in this review, two articles found a significant reduction in devices used per procedure (from 1.7 to 1.1 and 1.20 to 1.05, respectively), with shorter procedure times in the 3D-printed groups. Additionally, the 3D-printed models showed fewer devices deployed per procedure, perivascular leaks, and residual shunts. Additionally, one article found fewer perivascular leaks in the 3D group (one vs. four in controls), and one article showed no residual shunts in the 3D group compared to 14.29% in controls.

Although the 16 studies included in this review demonstrate the value of 3D printing in LAA occlusion procedures, the findings underscore the need for larger, multicenter studies to further quantify its clinical benefits, particularly in improving procedural planning and reducing complications in the intravascular treatment of LAA thrombosis in NVAF. Future research should focus on multicenter trials, larger cohorts, and testing 3D-printed occlusion devices for personalized treatments.

## Introduction and background

Patient-specific three-dimensional (3D)-printed anatomical models have been increasingly utilized in medicine for enhanced surgical preparation and trainee and patient education [1]. Left atrial appendage (LAA) occlusion is a crucial stroke prevention procedure for patients with nonvalvular atrial fibrillation (NVAF), as it reduces the risk of thromboembolic events. Accurate device sizing is critical to minimizing complications such as peridevice leaks and residual shunts, which can affect procedural success. In cardiology, 3D-printed models have been shown to help plan procedures for congenital heart diseases, such as aortic arch hyperplasia, Tetralogy of Fallot, and interventional procedures, such as transcatheter aortic valve replacement [1-4]. In these three procedures, 3D printing of the heart allows for simulated procedures to occur, enabling improved visualization of complex anatomy compared to two dimensions (2D). Models printed in different phases of the cardiac cycle can also allow the physician to appreciate the difference in the LAA size during systole and diastole [5].

3D printing can help model the LAA anatomy, thus facilitating more precise LAA occlusion and potentially reducing thrombus formation in patients with NVAF [6]. The use of flexible and durable materials, like elastic resin, a flexible, durable material used in 3D printing, enables the creation of highly detailed, patient-specific anatomical models. These models provide improved visualization of LAA morphology and facilitate more accurate device selection and placement. Additionally, the durability and the flexibility of this material are important because it can more accurately simulate the placement of the LAA occlusion device as opposed to a more rigid model. 

When oral anticoagulation is contraindicated, such as patients with history of bleeding or a high risk of bleeding, or not preferred by the patient, a procedure called LAA occlusion can be performed, where an occluder device is placed in the appendage to block clot formation under computed tomography (CT)/transesophageal echocardiogram (TEE) guidance [7-10]. Despite CT and TEE being the gold standard, both imaging methods have shown inaccuracies in measuring the size and volume of the LAA [3]. This can lead to procedural challenges, including the need for multiple device placements or adjustments. The physician may have to use multiple devices per procedure, perform additional procedures, or replace loosely fitting devices, which leads to increased costs and potentially improved patient outcomes.

**Figure 1:**
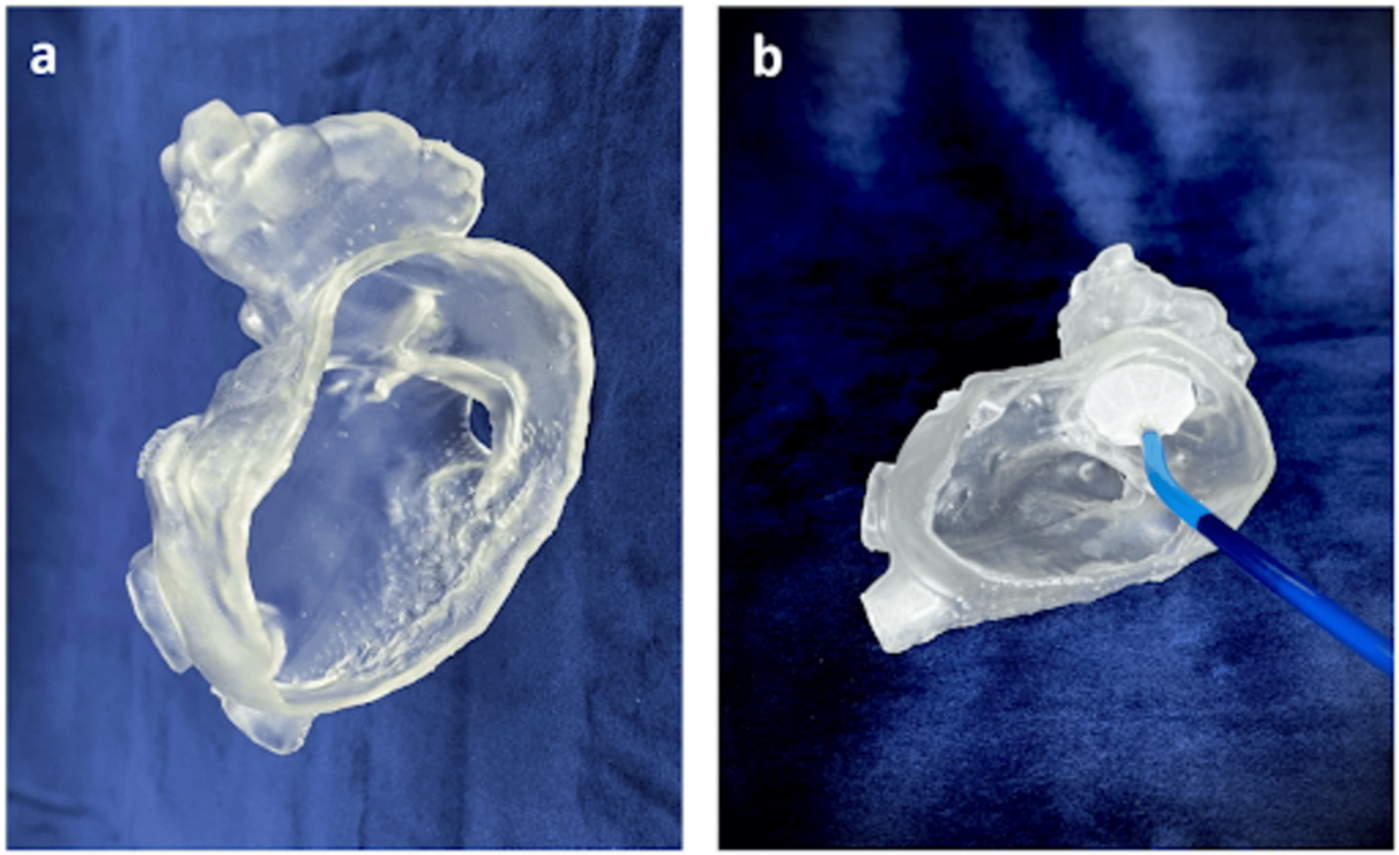
(a) 3D-printed anatomical model of the left atrium and LAA, printed using elastic resin on a Formlabs Form3B printer with a 2 mm-thick wall. (b) Same model with a Legacy Watchman™ device placed in the appendage to simulate occlusion LAA: left atrial appendage Image credits: Jasneel Kahlam

Figure 1 demonstrates the process of creating a 3D-printed anatomical model of the left atrium and LAA using volumetric data from CT. The model, printed with elastic resin on a Formlabs Form3B printer, features a 2 mm-thick wall designed to simulate the blood pool anatomy. This model was used to plan the deployment of a Legacy Watchman™ device, a commonly used LAA occlusion device, designed to seal the LAA and prevent clot formation. By simulating the procedure on the 3D model, clinicians can refine the sizing and placement of the occluder device, enhancing procedural accuracy and outcomes. The integration of these models into preprocedural planning provides a tangible and intuitive representation of complex cardiac anatomy.

**Figure 2:**
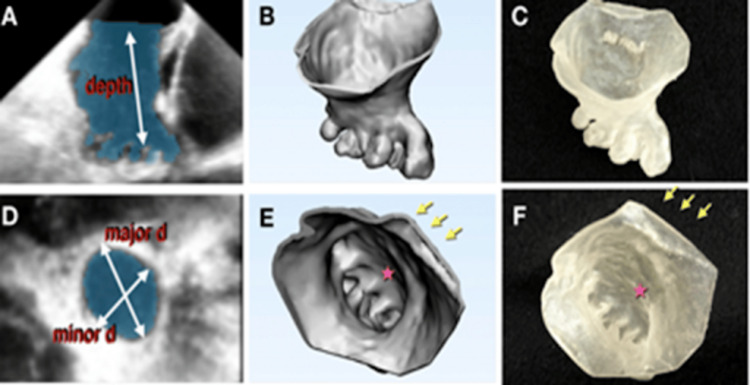
Progression of taking (a) echocardiography images to (b) 3D models to (c) 3D printouts. The same progression is depicted in (d) echocardiography images to (e) 3D models to (f) 3D printouts in a superior view Adapted/reproduced from Fan et al. [11], with open access permissions under the Creative Commons contribution agreement

Figure 2 illustrates the progression from echocardiographic imaging to the creation of 3D models and ultimately to 3D-printed replicas of the LAA. Images from Figure 2a to Figure 2f highlight examples of this workflow, demonstrating the transformation of imaging data into physical models that allow clinicians to evaluate anatomy in a tactile and spatial context. These models enable precise planning for LAA occlusion, providing critical insights into size and morphology during different cardiac phases. Such workflows underscore the utility of 3D printing in bridging imaging and procedural practice [11].

Since 3D printing is increasingly utilized for presurgical planning for LAA occlusion, this study systematically reviews the literature to assess the utility of using 3D printing to guide LAA occlusion procedures. Although other reviews have been conducted on this topic, most of them are about the cardiovascular field in general and do not include specific data on accuracy in device sizing and perivascular leakages. Two papers included data [4,5], but since their publication, numerous new studies have emerged.

One review included information about 3D printing for accurate fitting but failed to analyze the effect on perivascular device leakage and the number of devices per procedure [11]. A meta-analysis covered perivascular device leakage but did not analyze the number of devices used per procedure. In the same article, the systematic review also discussed perivascular leakage, the number of devices per procedure, and accurate fit but failed to analyze the type of anatomy or materials used for the models [12].

Overall, this review addresses significant gaps in the current literature, such as the lack of analysis on specific LAA anatomy variations and the materials used for 3D-printed models. Previous reviews have not adequately analyzed how anatomical variations influence procedural outcomes or how material choices for 3D-printed models impact device performance. These gaps are crucial as anatomical differences could affect the efficacy of 3D printing in occlusion procedures, and material choices might influence long-term device performance and patient outcomes. The question guiding this review is whether preoperative 3D printing can improve procedural outcomes and reduce complications in LAA occlusion, particularly in patients with varying anatomical profiles and using different occluder materials.

## Review

Methods

**Figure 3:**
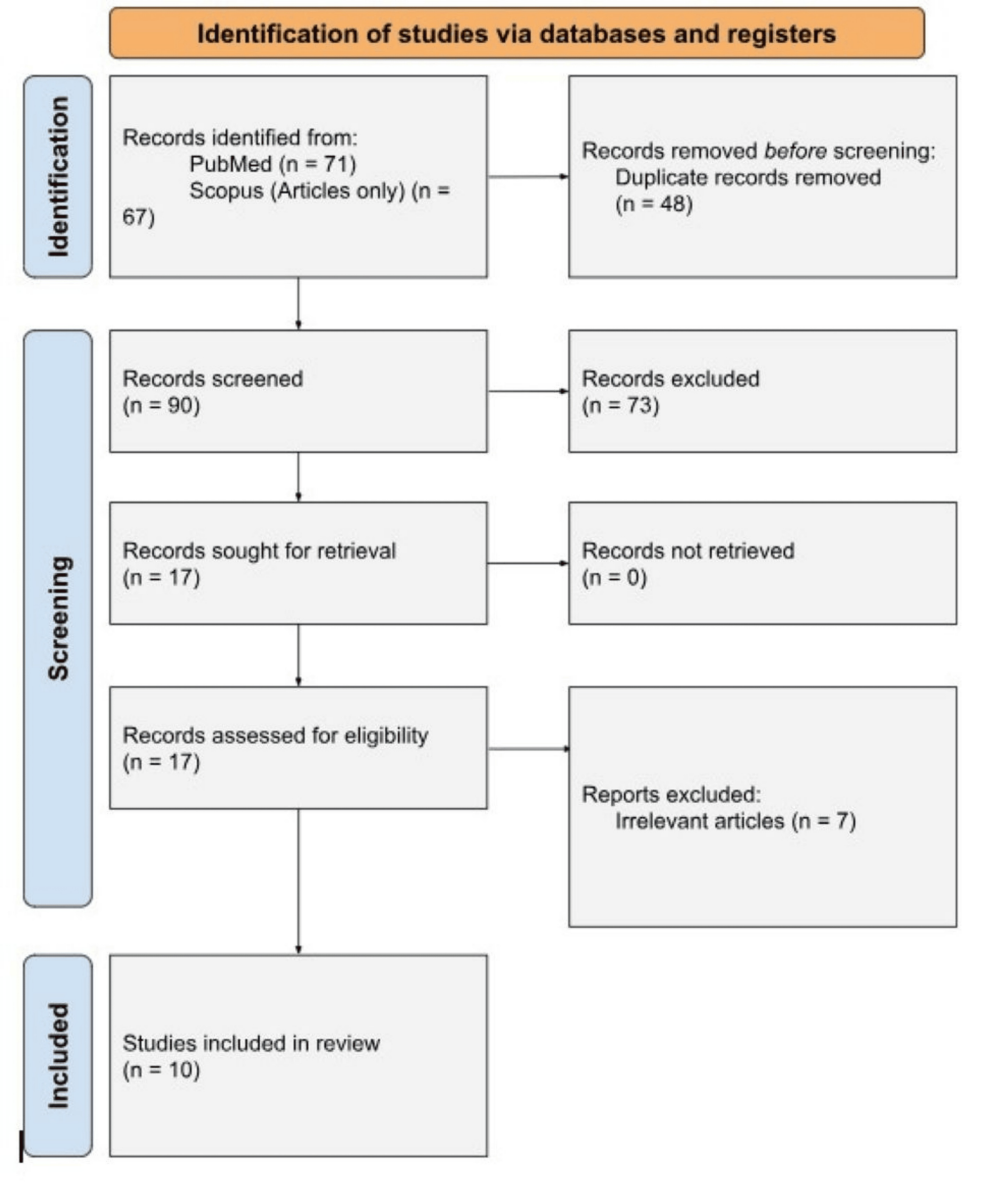
PRISMA flow diagram illustrating the screening and selection process for studies included in the analysis PRISMA: Preferred Reporting Items for Systematic Reviews and Meta-Analyses

This systematic review adhered to the 2015 Preferred Reporting Items for Systematic Reviews and Meta-Analyses (PRISMA) guidelines to ensure methodological transparency, rigor, and reproducibility [13]. A comprehensive search strategy was developed and executed to identify studies relevant to using 3D-printed models in facilitating LAA occlusion procedures. The review process encompassed detailed steps for inclusion and exclusion criteria, search strategy, data extraction, and synthesis. A PRISMA flow diagram (Figure 3) outlines the study selection process, including the number of records identified, screened, and included in the final analysis.

Search Strategy

A detailed electronic search was performed using PubMed and Scopus databases targeting literature published up to June 2024. Specific search terms included combinations of keywords and MeSH terms such as ("Left atrial appendage" OR "LAA" or “intra-atrial mass” OR “left atrial mass” ) AND ("3D printing" OR "additive manufacturing" OR "rapid prototyping" OR "computer-aided design" OR "CAD" OR "bioprinting") AND ("occlusion" OR "closure" OR "device sizing" OR "atrial fibrillation").

Two searches were conducted, the most recent of which was June 2024 to ensure the inclusion of recent studies (done by both J.K. and O.V.S). Only original articles written in English and focused on 3D printing applications for LAA occlusion procedures were considered eligible. The choice of January 2015 as the starting point was based on the significant technological advancements in 3D printing, particularly in the medical field, during this period. These advancements include improvements in printing resolution, material quality, and integration with imaging technologies, which have made 3D printing more applicable and reliable for preoperative planning in LAA occlusion. 

To ensure no relevant studies were missed, the update process involved setting up alerts in these databases for new publications related to 3D printing and LAA occlusion. Additionally, supplementary searches were conducted to capture any relevant studies that may have been overlooked in the initial search. This approach helped guarantee a thorough and up-to-date review of the literature.

Study Selection

A two-stage screening process was employed. In the first stage, titles and abstracts of all identified articles were reviewed independently by two researchers (J.K. and O.V.S.). Discrepancies were resolved through discussion and by a third author (D.F.L.). The mediation by a third author was conducted through a structured adjudication process where disagreements between the primary reviewers were resolved based on predefined criteria, such as the relevance of the study’s inclusion based on population, intervention, and outcomes. This was performed by a screening of abstracts and screening of full manuscripts on a program called Rayyan. In cases where disagreements persisted, the articles proceeded to a full-text review. 

To ensure a comprehensive review of the literature, the reference lists of included studies were also screened for additional relevant articles. In the second stage, full-text articles were independently assessed by the two authors, and any unresolved conflicts were mediated by the third author to ensure consistency in study selection. To minimize selection bias during the screening process, the screening was carried out independently by two reviewers, and discrepancies were discussed and resolved by the third.

Data Extraction and Synthesis

Relevant data were extracted from selected studies, focusing on using 3D printing techniques in LAA occlusion. The data extracted comprised several key components essential to assessing the impact of 3D printing in LAA occlusion procedures. First, study characteristics were manually documented, including the authors, year of publication, study design (such as prospective, retrospective, or randomized controlled trials), and sample sizes, as well as the study setting, whether it was conducted at a single center or multicenter. 

Patient demographics were also considered, with data on age, gender, and relevant pre-existing conditions or comorbidities, particularly atrial fibrillation or heart failure, which could influence the outcomes of LAA occlusion procedures. Details about the intervention were extracted, focusing on the specific 3D printing technology and materials used, as well as how the anatomical models were created, such as from CT or magnetic resonance imaging (MRI), and the complexity of the LAA as modeled. 

The primary outcome measures, including procedural time, the number of devices used per procedure, device leakage rates, and the presence of residual shunts, were recorded to quantify the effects of 3D printing on procedural efficiency and success. Additionally, data comparing 3D printing-assisted models to standard-of-care modalities, like CT or TEE imaging, were analyzed for accuracy in device sizing and the incidence of complications. 

Lastly, statistical analyses such as p-values, confidence intervals, and effect sizes (when available) were extracted to assess the significance of the findings. These elements were crucial in providing a comprehensive evaluation of 3D printing’s role in improving patient outcomes and procedural efficiency in LAA occlusion, compared to conventional imaging techniques. Any discrepancies in the extracted data were resolved through discussion and consensus with a third reviewer, ensuring reliability and consistency in the collection process. Figure 3 illustrates the detailed screening and selection process with no other software or programs used.

Inclusion Criteria

Studies published between January 2015 and June 2024 in English with full-text availability were included. All studies involved human subjects, were peer-reviewed, and focused on comparisons of 3D-printed models with TEE or CT imaging. Additionally, studies assessing perivascular or device leakages, the number of devices used per procedure, and comparisons between 3D-modeled occluder sizes and actual occluder sizes were evaluated. The included study designs encompassed cross-sectional studies, double-patient case studies, case-control studies, randomized controlled trials (RCTs), as well as prospective and retrospective studies.

Exclusion Criteria

Studies that lacked accessibility, were not involving human subjects, were non-English, were unavailable in full text, or were not peer-reviewed were excluded. Studies were excluded based on small sample sizes (less than eight patients), incomplete or missing data, methodological limitations such as lack of control groups or nonrandomized designs, and studies without a clear focus on 3D printing in LAA occlusion procedures. 

Additionally, studies unrelated to 3D-printed models compared with TEE/CT imaging, perivascular issues, or device leakages were omitted. Excluded formats included review articles, qualitative studies, single-patient case reports, editorial letters, meeting abstracts, gray literature, theses, crossover studies, ongoing clinical trials, and conference papers. Conference papers were excluded because they often present preliminary findings without the full validation and reproducibility required for rigorous analysis. Including such studies could introduce potential biases due to their preliminary nature and lack of comprehensive data.

Results

Using the Rayyan program, the key search terms used were 3D printing, left atrial appendage occlusion, computed tomography, TEE, device per procedure, and perivascular leakage. On the initial search, there were 92 references, 12 of which were eliminated as duplicates. Of the 80 remaining articles, 17 fit the inclusion criteria mentioned above. Ten articles were included after screening for full text.

In total, 10 articles met the inclusion criteria and were analyzed in this study [6,14-22]. Table 1 provides detailed information on each included study, including publication date, study design, and imaging type. Seven of the studies focused exclusively on printing the LAA without including adjacent anatomical structures [6,15,20-23]. Five studies included the left atrium (LA) and pulmonary veins in their 3D models [4,18]. Table 2 lists these studies and describes the 3D printing techniques utilized.

**Table 1:** Breakdown of each study: when published, design of study, and imaging type *: also includes 2D comparison; 3Dp: 3D printing; CT: computed tomography; TEE: transesophageal echocardiography; CTA: computed tomography angiography; RCT: randomized controlled trial; Ret: retrospective; Pro: prospective

Study	Study design	3D printing imaging type
Liu et al., 2016 [6]	Pro	TEE
Conti et al., 2019 [14]	Case-control study	CT
Fan et al., 2019 [11]	Pro/Ret	TEE
Hell et al., 2017 [16]	Pro	CT
Li et al., 2017 [17]	Pro, RCT	CTA
Obasare et al., 2018 [18]	Pro	CT
Wang et al., 2016 [19]	Pro	CT
Hachulla et al., 2018 [20]	Pro	CT
Hong et al., 2022 [21]	Pro	CT, MRI
Hozman et al., 2023 [23]	Pro	CT

**Table 2:** Further breakdown of each study: number of subjects, occluder type, effects of 3D printing on size estimates, and other parameters TEE: transesophageal echocardiography; CT: computed tomography; WM: watchman; AM: amulet; Ret: retrospective; Pro: prospective

Study	Number of subjects	Occluder type	3D printing effects on device size (compared to standard)	Other parameters
Liu et al., 2016 [6]	8	WM	Accurate size prediction: 8/8	n/a
Conti et al., 2019 [14]	20	Amplatzer cardiac plug	Device sizing: Underestimation: 11/20 Agreement: 7/20 Overestimation: 2/20 Predicted 75% accuracy with 3DP	n/a
Fan et al., 2019 [11]	Group 1: 72 (ret) Group 2: 32 (pro)	WM	Accurate prediction of size: 31/32. Corrected by 3D printing: 20/72	Better implantation rates, lower procedural times without complications
Hell et al., 2017 [16]	22	WM	Accurate prediction: 21/22	n/a
Li et al., 2017 [17]	42	WM	3D printing closer to actual size: 27.4 mm vs 27.6 mm	Three mild residual shunts in the control group. Reduced radiographic exposure
Obasare et al., 2018 [18]	22 (13 had 3Dp)	WM	14/14 (r^2^=1)	Less procedural time, less anesthesia time, less fluoroscopy time
Wang et al., 2016 [19]	53	WM	Perfect fits: 53/53	3D printed models saved time efficiency (48 ± 11) (first three patients). Adverse events in the first seven days: 0/53. Serious pericardial effusion within seven days: 0/53
Hachulla et al., 2018 [20]	15	Amulet cardiac plug	All 15 had accurate sizing: 3D TEE 8/15 and 3D CT 10/15	n/a
Hong et al., 2022 [21]	10	Not specified	Accurate prediction: 10/10	n/a
Hozman et al., 2023 [23]	60	Amulet, watchman	Accuracy: 55/60	n/a

Device Sizing

Of the 10 articles included in this analysis, five (50%) demonstrated that 3D-printed models could precisely predict the actual device size required for LAA occlusion with complete accuracy [6,17,18,20,22]. Three articles (18.8%) reported a 90% accuracy rate, meaning that in 90% of cases, the 3D-printed models predicted a device size within clinically acceptable error margins [14,16,22].

Additionally, three (30%) articles indicated that 3D-printed models showed improved procedural outcomes and reduced complications when compared to the standard-of-care imaging techniques (such as TEE or CT) [15,20]. Table 3 summarizes the impact of 3D printing on device size selection. The addition of models such as pulmonary veins and LA was used to simulate the left atrium anatomy, giving simulators a more accurate experience when placing the occlusion device into the appendage.

**Table 3:** The anatomy included in the 3D printed LAA models with a description of printing technologies and materials for each LA: left atrium; LAA: left atrial appendage; PV: pulmonary vein; SVC: superior vena cava; IVC: inferior vena cava

Name of study	Type of anatomy	3D printer	Printing material
Liu et al., 2016 [6]	LAA only	Stratasys Objet 30 pro 3D printer	Rubberlike material
Conti et al., 2019 [14]	Vena contracta of the LAA	Form 2 desktop printer	Vat-photopolymerization
Fan et al., 2019 [11]	LAA, left pulmonary ridge, part of the LA	Stratasys Objet350 Connex3 Polyjet printers	Rubber, translucent photopolymer material
Hell et al., 2017 [16]	LAA, left superior PV, proximal segment of the LAA	Ultimaker 2+ 3D printer	Two-component silicone rubber vulcanized
Li et al., 2017 [17]	LAA only	Chongqing Yingtaidike Technology Co	Does not specify
Obasare et. al., 2018 [18]	LAA only	Form 1+ desktop SLA printer	Does not specify
Wang et al., 2016 [19]	LA, LAA, Aortic annulus, rims of SVC and IVC	N/A	Does not specify
Hachulla et al., 2019 [20]	LA ( no interatrial septum)	Stratasys Objet260 Connex3 printer	Tangoplus FLX 930
Hong et al., 2022 [21]	LAA	Ultimaker BV, X Fab	TPU 95A, Flexa693

Device and Procedural Efficiency

One study (10%) showed that there was a significant decrease in the number of devices needed per procedure [16] Fan et al. reported that the prospective group that used 3D printing used significantly fewer devices per procedure (1.1 device per procedure) compared to the retrospective group (1.7 devices per procedure) [16] with a p-value < .0001.

Procedural outcomes: Two of the ten analyzed studies (20%) show that the cases of perivascular leaks and residual shunts are decreased when a 3D-printed model is used intraoperatively [18, 19]. In each of the studies, the prevalence of post-procedural perivascular leaks and residual shunts was used to assess how well the devices worked after the LAA placement. Obasare et al. reported peri-device leakages in four of the nine patients (44.4%) with standard-of-care procedures, but only one leak was found out of the 13 patients (7.7%) in the 3D printing-assisted group, with a p-value of 0.04 [19]. Table 4 compares the outcomes of 3D printing and standard of care for LAA occlusion planning.

**Table 4:** Outcomes compared between 3D printing assisted and standard of care LAA occlusion planning for device per procedures and device leakage TEE: transesophageal echo; CT: computed tomography; Ret: retrospective; Pro: prospective

Article citation	Type of Study	Imaging	Device per procedure	Device leakages
Fan et al., 2019 [11]	Pro/Ret	3D Printed TEE	1.1	n/a
Li et al., 2017 [17]	Pro, randomized control study	3D printed CTA imaging	n/a	3 mild residual shunts in control group
Obasare et al., 2018 [19]	Pro	3D printed CT imaging	n/a	Control: 4/19 3D printed: 1/13

Risk of Bias

**Figure 4:**
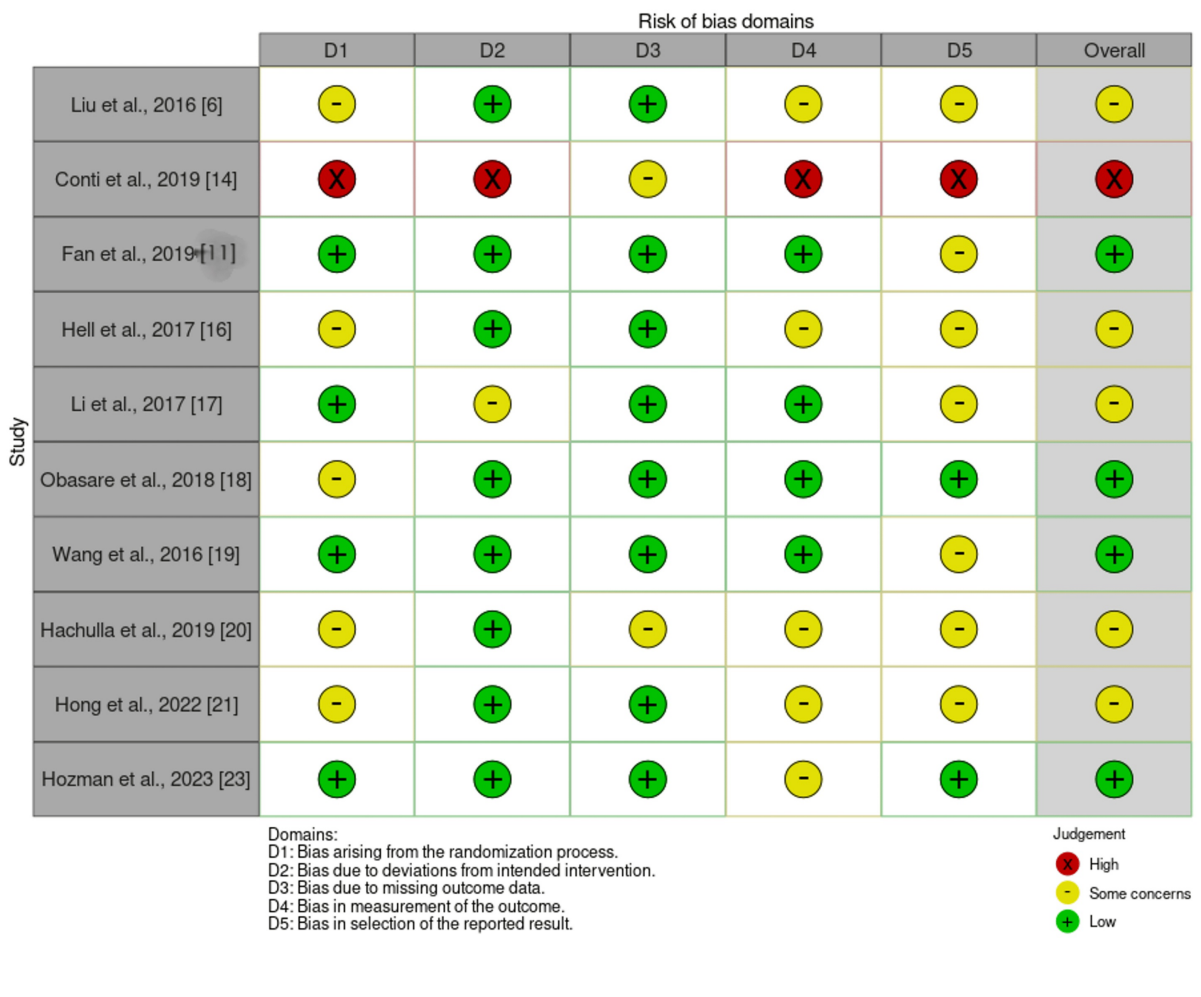
Cochrane risk-of-bias 2 assessment for included studies

The Cochrane Risk of Bias tool was used to assess the quality and potential biases in the included studies, focusing on five key domains: randomization process, deviations from intended interventions, missing outcome data, measurement of outcomes, and selection of reported results. Each study was evaluated based on these criteria, with the risk of bias classified as low, some concerns, or high. This tool enhances the transparency and rigor of the review by systematically identifying and documenting potential sources of bias. By incorporating this tool, the review provides a more robust understanding of the evidence quality, which is crucial for interpreting the results and guiding future research in the field of 3D printing for LAA occlusion, as shown in Figure 4.

Discussion

This review analyzed 10 studies that investigated how 3D-printed anatomical models have helped improve the visualization of LAA occlusion repair. The research described in this analysis demonstrated that 3D-printed models reduced post-procedural leakages and shunts, decreased the number of devices needed per procedure, and reduced complications due to inaccurate occluder sizing. These studies highlight that simulations or pre-surgical planning using 3D-printed models can help make procedures more efficient, thereby improving patient outcomes. In addition, since fewer devices were utilized with pre-operative 3D-printed models, the overall procedure became more efficient in terms of cost and time.

Another important benefit is the reduction in the number of devices required per procedure. Fan et al. reported that the number of devices used per patient decreased from 1.7 in the control group to 1.1 in the 3D printing-assisted group, representing a 35.3% reduction (p < 0.0001) [16]. This decrease not only minimizes procedural complexity but also contributes to cost savings by reducing the need for multiple device deployments. While hospitalization duration was not consistently reported in the reviewed studies, the combination of shorter procedure times, fewer complications, and improved device fit suggests that 3D printing could indirectly lead to shorter hospital stays, an area that warrants further investigation in future studies.

Beyond procedural efficiency, the ability of 3D-printed models to provide patient-specific anatomical accuracy plays a crucial role in optimizing device selection and positioning. Several studies indicated that 3D-printed models improve preoperative planning by offering an accurate physical representation of the LAA’s morphology, which is particularly valuable in cases with complex anatomical variations. This level of precision reduces the likelihood of suboptimal device placement, thereby improving long-term procedural success. Moreover, training and simulation using 3D-printed models may enhance the learning curve for new operators, as they allow for hands-on practice before performing the procedure on a patient.

Finally, a qualitative analysis of the included studies highlights both the benefits and challenges of integrating 3D printing into LAA occlusion planning. Many studies emphasize improved visualization and anatomical understanding, boosting clinician confidence in device selection and positioning, which reduces procedural adjustments and complications. The ability to physically manipulate patient-specific models also enhances preoperative planning, particularly for complex LAA anatomies. However, challenges include additional time and costs, variability in model accuracy based on imaging modalities, and a learning curve in effectively using the models. These qualitative factors are essential for evaluating the real-world feasibility of 3D printing in clinical practice alongside quantitative outcomes.

Future directions

Despite promising results, further studies are needed to confirm the benefits of 3D printing in planning intravascular LAA occlusion procedures. Most prospective studies reviewed had small patient populations, with only two of the 10 studies having more than 50 patients [19,23] and only one being a multicenter trial [16]. Larger, multicenter studies will allow for better examination of device leakage and the use of multiple devices per procedure. Future studies should also focus on testing the long-term durability of 3D-printed occluder devices.

Key unknowns regarding long-term durability include the structural integrity of devices, the risk of late peridevice leaks, and complications like device migration or thrombus formation. These complications could potentially undermine the advantages gained from improved preoperative planning. It's also unclear whether the improved anatomical fit via 3D-printed planning leads to sustained clinical benefits, such as reduced stroke risk or fewer device-related complications over time. As such, more research is necessary to assess the long-term clinical impact and validate the efficacy of 3D printing technology. More studies are also needed to study the effect on procedural cost, both short-term and long-term, as pre-planned operations can potentially cut down on fluoroscopy time and devices needed.

In the future, 3D printing could create personalized occlusion devices that are a better fit for the appendage. However, these devices must be biocompatible, durable, and immune system-friendly. While animal models have shown 3D-printed implants' feasibility [23], regulatory and ethical challenges complicate their use in humans. Patient-specific 3D-printed implants require extensive clinical trials to meet safety standards set by regulatory bodies like the FDA or EMA, and there have been no human studies on their insertion yet.

Beyond regulatory approval, practical challenges include manufacturing scalability, biocompatibility, and cost-effectiveness. The production of 3D-printed occluders must be efficient and reproducible while maintaining quality standards. Biocompatibility remains critical to prevent adverse immune responses, and cost-effectiveness needs evaluation to determine whether the benefits outweigh production costs. Although 3D printing could lower procedural costs by reducing the need for multiple devices, its overall economic impact on healthcare systems must be assessed through cost-benefit analyses and long-term financial models.

Limitations

A key limitation of this study is the small number of multicenter trials, which limits the generalizability of the findings. Only two studies in this review were multicenter, and such trials are essential for validating the broader applicability of 3D printing in LAA occlusion across different clinical settings [14, 16]. More multicenter trials would strengthen evidence for the role of 3D printing in improving device placement accuracy. Additionally, the lack of long-term data comparing 3D-printed and non-3D-printed device placements highlights the need for future studies to confirm the sustained benefits of 3D printing, particularly regarding device fit and procedural success.

Another limitation of the reviewed studies is the absence of variability data, such as standard deviations or interquartile ranges for device use per procedure. Without this data, it is difficult to assess the consistency and precision of 3D printing outcomes across different patient populations and clinical scenarios. While promising trends were noted, the lack of variability data prevents definitive conclusions about the robustness and reproducibility of 3D printing in LAA occlusion.

The review's reliance on PubMed, while efficient due to its extensive coverage of peer-reviewed medical research, is another constraint. Although PubMed provided high-quality studies directly related to 3D printing in LAA occlusion, broadening the search to include additional databases in future updates could enhance the comprehensiveness of the review. Despite this limitation, PubMed remains a reliable source for the studies analyzed, ensuring the validity of the review’s conclusions.

Moreover, procedural outcomes in the reviewed studies may be influenced by factors beyond 3D printing itself. Variables such as operator expertise, device selection, and imaging techniques could affect the success of the procedure. While some studies controlled for operator variability, others did not, and anatomical complexities may have independently impacted outcomes. To isolate the true effect of 3D printing on LAA occlusion, standardized protocols and larger multicenter studies are needed.

Additionally, publication bias is a concern that could potentially overstate the benefits of 3D printing. To minimize this risk, future research should adopt pre-registered protocols and apply rigorous statistical methods to ensure objectivity. The lack of standardization in 3D printing techniques and materials across studies further complicates reproducibility and comparability. Establishing standardized protocols for 3D printing would improve the consistency of future studies and enable more reliable comparisons.

Finally, retrospective studies, which were prevalent in the reviewed literature, may be subject to selection bias due to their reliance on previously collected data. This data may not accurately reflect the broader patient population. While prospective studies can offer more controlled data collection and more accurate insights, they, too, can introduce biases related to participant selection and variability in the procedures themselves. As such, both retrospective and prospective studies have limitations, underscoring the need for more robust research designs to assess the true impact of 3D printing on LAA occlusion.

## Conclusions

This systematic review highlights the significant potential of 3D printing technology in planning LAA occlusion procedures. The analyzed studies demonstrate that 3D-printed models enhance procedural accuracy by improving anatomical measurements, optimizing device sizing, reducing the number of devices per procedure, and minimizing perivascular leaks and residual shunts. These benefits not only improve patient outcomes but also enhance procedural efficiency, underscoring the value of integrating 3D printing into preoperative workflows.

Despite these promising findings, limitations such as small sample sizes, limited multicenter trials, and a lack of long-term outcome data limit the current evidence for broader adoption. Future research should focus on larger, multicenter, and longitudinal studies to validate these benefits and explore innovations like patient-specific 3D-printed occlusion devices. Creating personalized models of the LAA occluder devices could potentially increase accuracy in device sizing as well as prevent complications such as perivascular shunts. Additionally, registries tracking patient outcomes, such as strokes, mortality, and device thrombosis, over extended periods could provide valuable data on whether 3D printing improves long-term clinical success compared to conventional planning methods. Such advancements could further personalize and refine LAA occlusion procedures, optimizing care for patients with NVAF.
